# First Whole Genome Sequence of *Anaplasma platys*, an Obligate Intracellular Rickettsial Pathogen of Dogs

**DOI:** 10.3390/pathogens9040277

**Published:** 2020-04-10

**Authors:** Alejandro Llanes, Sreekumari Rajeev

**Affiliations:** 1 Centro de Biología Celular y Molecular de Enfermedades, Instituto de Investigaciones Científicas y Servicios de Alta Tecnología (INDICASAT AIP), Ciudad del Saber, Panama City 0801, Panama; allanes@indicasat.org.pa; 2 School of Veterinary Medicine, Ross University, Basseterre KN 0101, Saint Kitts and Nevis

**Keywords:** *Anaplasma platys*, dogs, whole genome sequence, tick-borne pathogens

## Abstract

We have assembled the first genome draft of *Anaplasma platys*, an obligate intracellular rickettsia, and the only known bacterial pathogen infecting canine platelets. *A. platys* is a not-yet-cultivated bacterium that causes infectious cyclic canine thrombocytopenia, a potentially fatal disease in dogs. Despite its global distribution and veterinary relevance, no genome sequence has been published so far for this pathogen. Here, we used a strategy based on metagenome assembly to generate a draft of the *A. platys* genome using the blood of an infected dog. The assembled draft is similar to other *Anaplasma* genomes in size, gene content, and synteny. Notable differences are the apparent absence of *rbfA*, a gene encoding a 30S ribosome-binding factor acting as a cold-shock protein, as well as two genes involved in biotin metabolism. We also observed differences associated with expanded gene families, including those encoding outer membrane proteins, a type IV secretion system, ankyrin repeat-containing proteins, and proteins with predicted intrinsically disordered regions. Several of these families have members highly divergent in sequence, likely to be associated with survival and interactions within the host and the vector. The sequence of the *A. platys* genome can benefit future studies regarding invasion, survival, and pathogenesis of *Anaplasma* species, while paving the way for the better design of treatment and prevention strategies against these neglected intracellular pathogens.

## 1. Introduction

Members of the genus *Anaplasma* are classified in the *Anaplasmataceae* family within the order *Rickettsiales* of class Alphaproteobacteria. The family *Anaplasmataceae* encompasses several species of obligate intracellular bacterial pathogens of human and animal health significance, listed in the *Anaplasma*, *Ehrlichia*, *Wolbachia*, and *Neorickettsia* genera. They are small, pleomorphic, coccoid to ellipsoidal cells, 0.3–0.4 μm in diameter, found in cytoplasmic vacuoles of mammalian host cells as inclusion bodies (morulae). Members of this family have complex life cycles involving vertebrate hosts and invertebrate vectors, and many of them are emerging vector-borne pathogens. Clinically relevant members of the *Anaplasma* genus include *A. marginale, A. centrale, A. ovis, A. phagocytophilum,* and *A. platys.* They are transmitted by multiple species of hematophagous ticks and, in the infected vertebrate hosts, they effectively colonize leukocytes or other bone marrow-derived cells, potentially leading to diverse clinical presentations collectively known as anaplasmosis.

*Anaplasma platys* was first reported in 1978 as a *Rickettsia*-like bacterium causing thrombocytopenia in dogs and was later identified as the causative agent of infectious canine cyclic thrombocytopenia [1,2]. Both *A. platys* and *A. phagocytophilum* were originally placed in the *Ehrlichia* genus and were later reclassified as *Anaplasma* [3]. In infected dogs, parasitemia and thrombocytopenia are reported to be cyclic, occurring at frequent intervals of 8–15 days, possibly becoming fatal due to severe thrombocytopenia and subsequent hemorrhage. *A. platys* is suspected to be transmitted through the brown dog tick *Rhipicephalus sanguineus*, a ubiquitous member of the ixodid tick family, and the infection is frequently presented as a comorbidity with *Ehrlichia canis*, another rickettsial pathogen transmitted by the same vector. *A. platys* is the only known bacterial pathogen that exclusively infects platelets, small anucleated bone marrow-derived cellular components responsible for hemostasis. The significance of platelets in infection, inflammation, and immunity has increasingly been acknowledged [4,5]. Very little is known about the pathogenesis and attributes of this pathogen that infects a critical cellular component in the blood such as platelets. Zoonosis is also suspected, with a few cases reported in humans [6,7].

Whole genome sequencing of obligate intracellular pathogens has been hindered by the difficulty in growing them in laboratory media, since the isolation of the pathogen’s genomic DNA has to be done in the presence of host DNA. The sequencing of the *A. marginale* genome was initially achieved by using the laborious and expensive strategy of creating bacterial artificial chromosome (BAC) libraries of bacterial and host DNA with minimal purification of the pathogen from the host cell, followed by the selection of clones containing bacterial genes [8]. Subsequent genomic studies, involving *Anaplasma* isolation, used dedicated laboratory procedures such as culturing the bacteria in vitro in host cells [9] or cells from the tick vector [10] or inoculating the original isolates in splenectomized animals in order to obtain relatively high levels of bacteremia [11,12]. Targeted enrichment of genomic DNA has also been used as an alternative to sequence *A. marginale, A. phagocytophilum* and *Wolbachia* genomes [13,14,15].

All of the *Anaplasma* genomes sequenced so far are composed of a single circular chromosome with a total size of 1–1.5 Mbp and ~900 protein-coding genes. The relatively small genome size in this genus appears to be associated with a process of reductive evolution that has occurred in the order *Rickettsiales* due to long-term intracellular association with eukaryotic hosts [16]. This reductive evolution is associated with the frequent formation of pseudogenes, affecting distinct loci in different species [17]. Several complete genome sequences have been published for *Anaplasma* spp., but no genome has been sequenced so far for *A. platys*. In this study, we used enrichment methods to concentrate *A. platys* from the blood of a clinically infected dog and further applied a strategy based on metagenome assembly to generate a draft of the *A. platys* genome. We report several comparative analyses of the *A. platys* genome with previously published genomes of *Anaplasma* and other related species.

## 2. Results

### 2.1. Sample Collection and DNA Extraction

The canine patient in this study was presented to the Ross University Veterinary Clinic, Basseterre, Saint Kitts, West Indies. The patient presented extreme lethargy, anorexia, and pale mucous membranes. A complete blood count (CBC) revealed leukopenia, neutropenia, and thrombocytopenia. On examination of the blood smear, we observed morulae in platelets, compatible with *A. platys* infection (Figure S1). *A. platys* infection was further confirmed by polymerase chain reaction (PCR). DNA extracted after enriching and concentrating infected blood components resulted in successful retrieval of the *A. platys* genome from the sample.

### 2.2. Genome Assembly and Annotation

Sequencing of total DNA extracted from the canine blood sample resulted in more than 53 million 150-bp Illumina reads, of which 96% were unambiguously aligned to the canine reference genome and were further discarded. The remaining unmapped reads were assembled de novo with metaSPAdes [18]. A *k*-mer size of 55 was chosen for de novo assembly after comparing metrics for tests performed with *k*-mer sizes of 21, 33, and 55. Sixty-five scaffolds with at least one BLAST hit to previously published *Anaplasma* sequences were pooled together to generate the first tentative assembly of the *A. platys* genome, with an *N*_50_ value of 32 kb.

In order to identify potential assembly artifacts, original reads that did not map to the canine reference genome were mapped against the assembled scaffolds. Alignments were visually checked in order to detect any anomalous distribution of mapping pairs with an estimated distance different from that expected between read mates. Read depth along the scaffold sequence was relatively uniform for most scaffolds, except for scattered peaks associated with multicopy gene families (discussed below). However, two of the original scaffolds were split into five fragments based on suspected assembly artifacts. ABACAS [19] was further used to order and orient the split fragments and the rest of the scaffolds into a single pseudochromosome using the *A. phagocytophilum* genome as a reference. This strategy differs from a reference-guided assembly in that it uses the scaffolds instead of the reads, thus retaining the particular genome arrangements that were assembled de novo.

Two strategies were used to annotate the newly assembled *A. platys* genome draft, based respectively on de novo gene identification and comparative transfer of gene models previously annotated in the *A. marginale* and *A. phagocytophilum* reference genomes. After manual revision and curation, the assembled pseudochromosome has a total size of 1.2 Mbp, 1.8% of ‘N’, and 869 putative coding sequences (CDSs), 13 of which were flagged as putative pseudogenes. These basic metrics are similar to those from previously published *Anaplasma* genomes (Table 1). The GC percent calculated for our *A. platys* genome draft is relatively lower than that for *A. marginale* or *A. ovis*, a finding that has also been reported for *A. phagocytophilum* [9,12]. The annotated genome was deposited in the GenBank database with accession number CP046391, under BioProject PRJNA578763.

**Figure 1:**
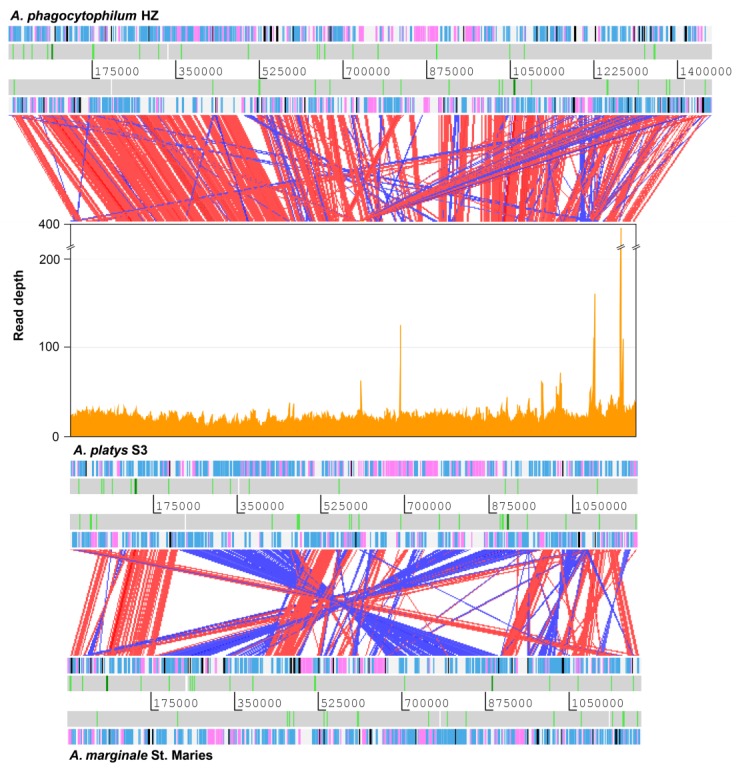
Comparison of the *A. phagocytophilum*, *A. platys,* and *A. marginale* genomes. Coverage of Illumina reads used for de novo assembly is plotted directly above the *A. platys* genome. Protein-coding genes are colored blue (function known/inferred) and pink (function unknown), while pseudogenes are colored black. RNA genes are represented as white bars with rRNA and tRNA genes highlighted in dark green and light green, respectively. Diagonal bars across genomes indicate sequence similarity matches in the forward (red) or reverse (blue) direction. Bars radiating from the *A. platys* genome to that of *A. phagocytophilum* indicate matches to genes/pseudogenes of the *msp2* superfamily, highly expanded in the latter.

Genome comparison at the nucleotide level revealed relatively high sequence divergence among all *Anaplasma* and *Ehrlichia* genomes considered in this study, including our newly assembled *A. platys* genome. However, comparison at the level of translated reading frames, by using the tblastx program of the BLAST suite, showed reasonable sequence similarity between coding regions (Figure 1). Based on these results, our assembly was found to cover 98% of the non-repetitive part of the *A. phagocytophilum* genome. Similar results were obtained when comparing our draft assembly with the *A. marginale* genome, although in this case, sequence similarity among coding regions was generally lower and there were more large-scale genome rearrangements. In contrast, our assembly is more similar in size to the *A. marginale* genome than to that of *A. phagocytophilum*, in part due to the putatively higher number of genes from the *msp2* superfamily, highly expanded in the latter.

### 2.3. Genome Completeness and Phylogenetic Analysis

Since we used a metagenomic strategy to generate a draft of the *A. platys* genome, we were particularly interested in assessing the completeness of this draft. This was done with the BUSCO pipeline, which uses predefined lineage-specific sets of benchmarking universal single-copy orthologs (BUSCOs) as a reference for gene content expectations [20]. However, since all members of the order *Rickettsiales* have undergone a well-documented natural process of genome reduction, we included 18 representative species of this order, 15 from the *Anaplasmataceae* family (genera *Anaplasma*, *Wolbachia*, *Ehrlichia,* and *Neorickettsia*) and three from the *Rickettsiaceae* family (genus *Rickettsia*) (Table S1). *Escherichia coli* strain K12 was used as a reference since it was the best option among the reference lineages predefined in the pipeline. *E. coli* O157:H7 and *Neisseria meningitidis* were also included in the analysis for comparative purposes, since no significant genome reduction has been reported for these species.

**Figure 2:**
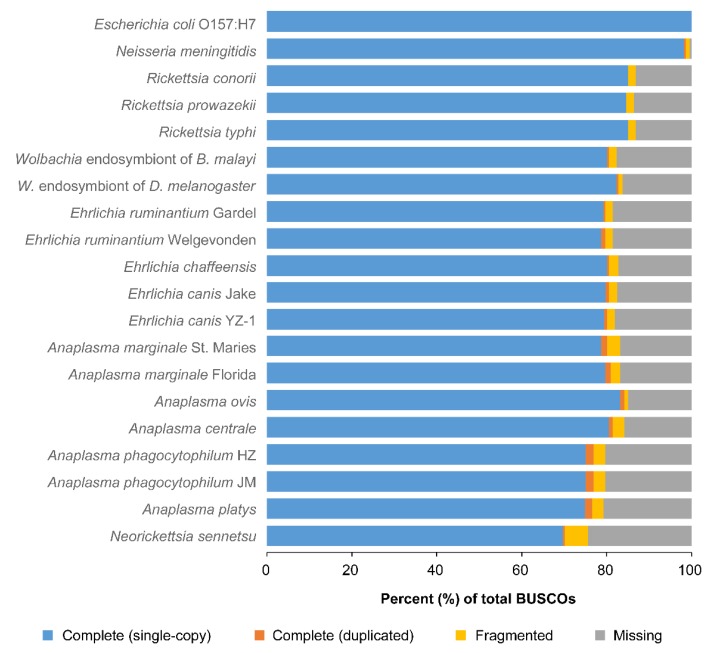
Assessment of genome completeness for *A. platys* and several other species of the *Rickettsiales* order. Completeness was assessed with the BUSCO pipeline, which uses lineage-specific ortholog sets of conserved genes called BUSCOs. For each species, bars show the percent of BUSCOs from the reference lineage that were unambiguously found in the genome either as a single copy or duplicated, as well as those that were only partially found or were missing.

Not surprisingly, the results showed a relatively lower percentage of completeness for all the rickettsial genomes when compared to those of *E. coli* O157:H7 or *N. menigitidis* (Figure 2). Genome completeness estimated for *A. platys* was similar to that of *A. phagocytophilum* strains HZ and JM, averaging 76%. These two species also showed some of the lowest percentages of completeness, second only to *N. sennetsu* (70%). All other *Anaplasma* species, as well as *Ehrlichia* and *Wolbachia*, had estimates equal to or slightly higher than 80%. *A phagocytophilum* also had the largest estimates of duplicated genes in BUSCOs, which is consistent with previous reports of extensive gene duplication in this species [9,10]. Results also suggest a relatively high degree of gene duplication in *A. platys*, comparable to that estimated for *A. phagocytophilum*.

**Figure 3:**
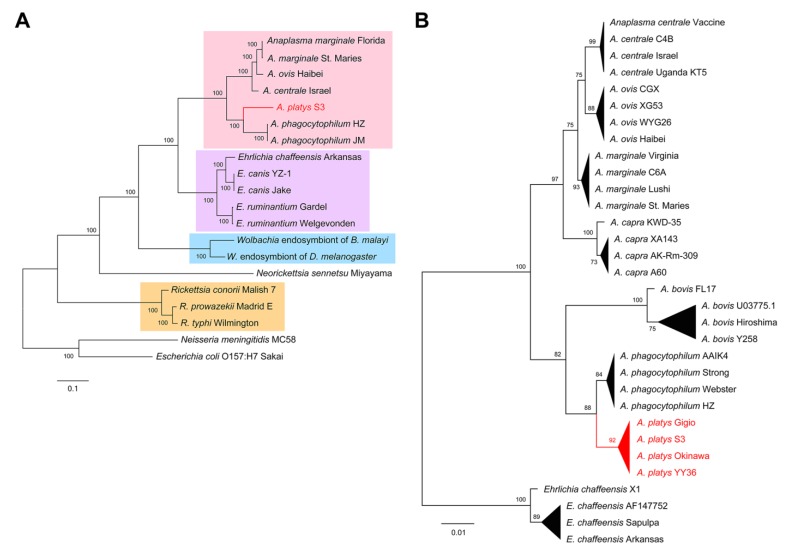
Evolutionary position of *A. platys*. (**A**) ML trees built from concatenated amino acid sequences of conserved genes from the species included in the genome completeness analysis (Figure 2). Branches grouping *Anaplasma*, *Ehrlichia*, *Wolbachia,* and *Rickettsia* species were respectively highlighted in pink, purple, blue, and orange. (**B**) ML tree built from the nucleotide sequences of 16S rRNA genes of four representative strains/isolates for seven *Anaplasma* species, using *E. chaffeensis* as an outgroup.

Since the assessment of genome completeness provides a catalog of gene conservation across species, we selected 27 conserved genes (Table S2) to explore the evolutionary position of *A. platys* in a strategy similar to that suggested by Dunning Hotopp et al. [9]. Amino acid sequences of these genes were concatenated, aligned, and used to build a maximum likelihood (ML) tree (Figure 3A). We also built an ML tree from nucleotide sequences of the 16S ribosomal RNA (rRNA) gene (Figure 3B), this time considering four strains/isolates for representative species of the *Anaplasma* genus and using *E. chaffeensis* as an outgroup (Table S3). Both trees support the close relatedness between *A. platys* and *A. phagocytophilum* and their placement in the *Anaplasma* genus. Conversely, *E. canis*, a species that is often found co-infecting dogs positive for *A. platys* [21], was clustered within the *Ehrlichia* branch. The rRNA tree also suggests that *A. bovis* is more closely related to *A. phagocytophilum* and *A. platys* than *A. capra*.

### 2.4. Comparative Analysis of Protein-Coding Genes

**Figure 4:**
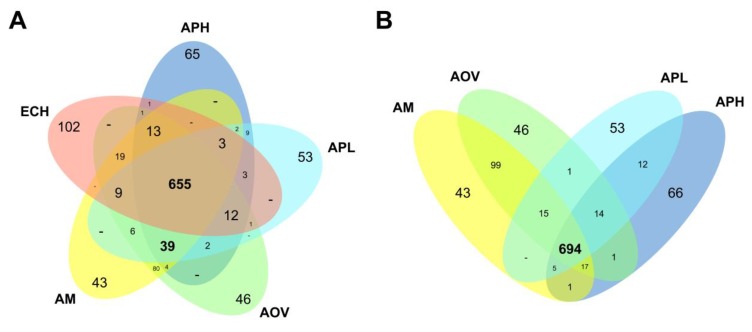
Differences in gene content among representative *Anaplasma* species and *E. chaffeensis*. Venn diagrams show the distribution of ortholog groups and potential species-specific genes among representative *Anaplasma* species, including *E. chaffeensis* (**A**) and excluding this species (**B**). The 664 groups shared by all species are detailed in Table S4. The remaining groups and species-specific genes are detailed in Table S5, using the colors of the Venn diagram in panel (A). AM: *A. marginale*, AOV: *A. ovis*, APH: *A. phagocytophilum*, APL: *A. platys*, ECH: *E. chaffeensis*.

In order to explore the differences in gene content among the *A. platys* genome and those from closely related species, we used OrthoMCL [22] and subsequent manual curation to cluster the protein-coding genes into putative ortholog groups. Annotated gene models from the *E. chaffeensis*, *A. marginale*, *A. ovis*, *A. phagocytophilum,* and *A. platys* genomes were clustered into 912 ortholog groups, of which 655 appear to be shared by the five species (Figure 4A; Table S4). The number of shared ortholog groups increased just slightly to 694 when not considering *E. chaffeensis* (Figure 4B), thus supporting the close relatedness of genera *Ehrlichia* and *Anaplasma*. Most of the groups or genes not globally shared by all *Anaplasma* species are likely to be species-specific (Table S5), with the notable exception of 99 groups shared between *A. marginale* and *A. ovis*.

Our ortholog clustering analysis roughly matches the analysis conducted by Dunning Hotopp et al. [9] for a broader range of species. Almost all the groups reported by the authors in conserved categories were among the 655 shared ortholog groups mentioned above and have at least one ortholog in *A. platys*, including those common to all obligate and facultative intracellular bacteria or those that are specific to *Rickettsiales* and *Anaplasmataceae*. A notable exception is a gene encoding the 30S ribosome binding factor RbfA (ortholog group OG_0718), shared by all intracellular bacteria but not found in our *A. platys* genome draft. To confirm the absence of this gene in strain S3, we searched for the sequences of the corresponding orthologs in the original Illumina datasets by using BLAST. We did not find any significant sequence similarity with the orthologs in the reads selected for de novo assembly or in the original reads before filtering those mapping the canine genome.

We found 13 potentially recent pseudogenes in the *A. platys* genome, harboring high-impact mutations present in the majority of Illumina reads mapping to the corresponding positions. Two of these genes encode enzymes involved in biotin metabolism, namely, dethiobiotin synthase (OG_0734) and biotin synthase BioB (OG_0728). Most of the remaining pseudogenes appear to be associated with genes in potentially expanded gene families, four of them putatively belonging to the *msp2* superfamily (ANPL_02540, ANPL_04015, ANPL_04156, and ANPL_04311). However, two pseudogenes appear to be part of multicopy gene clusters organized in short tandem arrays in *A. platys*, but their orthologs are apparently single-copy in the other four species (see Gene Duplication and Expansion of Gene Families). One of these genes, ANPL_0101, belongs to group OG_0021, encoding a cytosol aminopeptidase with two additional intact copies (ANPL_01015 and ANPL_01025). The other one, ANPL_02485, was clustered into group OG_0040, putatively encoding a transporter from the cation:dicarboxylate symporter family, also with two additional intact copies (ANPL_02480 and ANPL_02490).

We also found 53 potentially specific genes present in the *A. platys* genome. In agreement with previous studies, the vast majority of these putative species-specific genes encode proteins of unknown function, except for one of them belonging to the *msp2* superfamily (ANPL_02790) and another one encoding an ankyrin repeat-containing protein (ANPL_02687). Interestingly, four of the genes encoding proteins of unknown function (ANPL_03315, ANPL_03345, ANPL_03375, and ANPL_03280) have several predicted intrinsically disordered regions in the corresponding amino acid sequences, in some cases covering a significant portion of their sequence (Figure S2).

Despite their close relatedness, we found only 12 ortholog groups exclusively shared between *A. platys* and *A. phagocytophilum*, with three more also shared with *E. chaffeensis* but not with *A. marginale* or *A. ovis*. Genes in these shared groups appear to encode for another ankyrin repeat-containing protein (OG_0906), a vacuolar membrane-interacting protein (OG_0909), a putative RNA-binding protein (OG_0794), and DNA mismatch repair endonuclease MutL (OG_0798), among several other proteins of unknown function. The relatively low number of ortholog groups specific to *A. platys* and *A. phagocytophilum* contrasts with the higher number of genes that appear to be exclusively shared by *A. marginale* and *A. ovis*, including those belonging to the widely studied families such as *Anaplasma* appendage associated-proteins (*aaap*) or the *msp2* superfamily (discussed below).

Except for those involved in biotin metabolism, genes distinctively present or absent in *A. platys* do not seem to encode proteins associated with metabolic pathways. Consequently, the metabolic capacity of *A. platys* is likely to be similar to that previously discussed for other *Anaplasma* species [8,9,12], characterized by incomplete pathways for glycolysis and the synthesis of most amino acids. In fact, metabolic reconstruction of the *A. platys* strain showed that the pathways that appear to be complete are the central pathways of energy metabolism from pyruvate oxidation to the citric acid cycle and oxidative phosphorylation, the non-oxidative phase of the pentose phosphate pathway, as well as biosynthetic pathways for fatty acids, coenzyme A, glutathione, and nitrogenous bases (Table S6).

To further assess the conservation of gene order across the five species, we arbitrarily assigned a color in a green–blue gradient to each of the ortholog groups described above, ordered by the genes of the *A. marginale* genome. These colors were then plotted comparatively along the sequence of each genome according to their respective gene order (Figure S3). Results show that, despite all the obvious differences in gene content, synteny appears to be well-conserved across species, altered mainly by large-scale rearrangements and expansion of gene families. Gene order appears to be more similar for *A. phagocytophilum*, *A. platys,* and *E. chaffeensis* when compared to *A. marginale* and *A. ovis*.

### 2.5. Gene Duplication and Expansion of Gene Families

**Figure 5:**
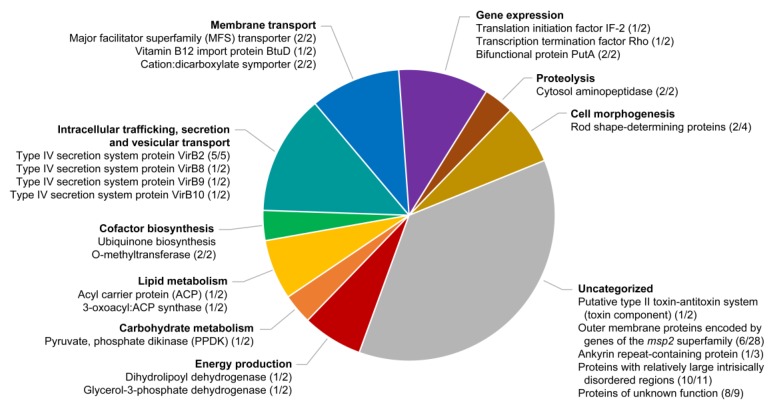
Functional categorization of duplicated genes or expanded gene families in the *A. platys* genome. The figure shows the putative products of genes clustered in different functional categories inferred from clusters of orthologous group (COG) assignments and gene ontology (GO) terms (see Materials and Methods). The two numbers separated by a slash (“/”) after each product indicate the respective number of copies that could be assembled de novo and those that were estimated from variations in Illumina read depth.

Assembly of duplicated genes or those expanded into gene families is typically difficult when using relatively short Illumina reads. These reads tend to collapse into one of the copies, particularly in cases when the duplicated genes are highly similar in sequence. Here, duplicated genes or expanded gene families that could be assembled de novo were identified by examining the ortholog clusters with more than one gene member, while those that could not be assembled due to read collapse were identified by local variations in Illumina read depth. Globally, we found more than 35 putatively duplicated or expanded genes in the *A. platys* genome, of which seven appear to have more than two copies (Figure 5, Table S7). Copy numbers estimated from variations in Illumina read depth suggest that there are at least 70 more protein-coding genes and three more tRNA genes in the genome, in addition to those that could be assembled de novo.

The *msp2* superfamily is the largest multicopy gene family, with six annotated gene members, eight fragments or potential pseudogenes, and 14 additional copies estimated from increased read depth, totaling 28 estimated members. Due to the previously mentioned technical limitations, we were not able to properly assemble many of these loci, therefore, we opted for a phylogenetic, rather than structural, analysis to preliminary characterize the genes present in *A. platys*. Although sequence divergence in this superfamily is too high to build robust phylogenetic trees, we used the neighbor-joining method to cluster representative members of the superfamily from *E. chaffeensis*, *A. marginale*, *A. ovis*, *A. phagocytophilum,* and *A. platys* (Figure S4). Genes found to be fragmented or truncated after sequence alignment were excluded from the analysis to simplify the results. The tree clearly separates paralogs from *E. chaffeensis* from those of *Anaplasma* spp. Within *Anaplasma*, genes were clustered into several clades, roughly consistent with previously reported subsets such as *msp2/p44*, *msp3*, *msp4*, *omp,* and *opag* [8,9]. Unambiguous assignment of individual genes into these subsets is difficult, but grouping patterns of *A. platys* paralogs in the tree revealed that, regardless of the subsets, these genes tend to be close to those from *A. phagocytophilum*, while paralogs from *A. marginale* are closer to those from *A. ovis*. As reported previously for *A. phagocytophilum*, we did not find an *msp3* ortholog in *A. platys*. However, the tree shows an expansion of *msp2/p44* genes in both *A. platys* and *A. phagocytophilum*, in a branch closely related to that of *msp3* orthologs from *A. marginale* and *A. ovis*. Furthermore, the tree suggests that some *omp* paralogs (*omp-4* to *10*) appear to have undergone a pattern of expansion in *A. marginale* and *A. ovis* comparable to that of *msp2/p44* in *A. phagocytophilum* and *A. platys*. These findings suggest that the corresponding gene lineages may have undergone selective expansion in these two pairs of species, in some cases leading to highly divergent paralogs.

Another example of an expanded family is that of genes encoding type IV secretion system protein VirB2 (OG_0003), which is expanded in *E. chaffeensis* and all other *Anaplasma* species. Also, another ankyrin repeat-containing protein (ANPL_02900) appears to be expanded in *A. platys*, but it is apparently single-copy in the other species. All other genes predicted to have more than two copies appear to encode proteins of unknown function and are likely to be specific to *A. platys*, except those clustered in ortholog group OG_0007. This group has one or two orthologs in all other *Anaplasma* species but not in *E. chaffeensis*. In *A. platys*, we found five copies of these genes, organized in a tandem gene array, together with genes ANPL_03315, ANPL_03345, and ANPL_03375 (OG_0755). Several members of OG_0007 also have predicted intrinsically disorder regions in their amino acid sequences (Figure S2).

In addition to expanded gene families, we also found several examples of strictly duplicated genes, i.e., those that have exactly two copies, most of which appear to be organized in tandem pairs or with the two copies located close together in the chromosome. Many of these genes encode proteins involved in metabolic pathways, membrane transport, gene expression, and cell morphogenesis. Genes involved in similar functions have been reported to be duplicated in other rickettsial genomes. However, although many of these strictly duplicated genes are also present in other *Anaplasma*/*Ehrlichia* species, most of their orthologs appear to be single-copy in these species. Two notable exceptions are genes from ortholog groups OG_0026 (dehydrolipoyl dehydrogenase) and OG_0761 (unknown function), which are also apparently duplicated in *A. phagocytophilum*. At least two duplicated genes, however, appear to be exclusively present in *A. platys*, both of them encoding proteins of unknown function.

## 3. Discussion

In this study, we successfully assembled and annotated a draft of the *A. platys* genome, starting with whole DNA extracted from an infected dog’s blood. Since *A. platys* is a not-yet-cultivated intracellular bacterium, we opted for a metagenomic approach for genome assembly. In this strategy, whole genome sequencing was used to sequence the entire DNA extracted from the infected blood components. Bioinformatic tools were then used to separate or capture the pathogen-derived sequences. Since this methodology could potentially lead to improper assembly completeness and contamination with foreign DNA, we performed several steps to assess proper completeness and to minimize and check for contamination. Globally, our validated *A. platys* genome draft is similar to other *Anaplasma* genomes in size, gene content, synteny, and the presence of duplicated genes and expanded gene families. Comparison of the *A. platys* assembly with the reference genomes of *A. marginale* and *A. phagocytophilum* revealed a reasonably good coverage of the non-repetitive portions of those genomes (up to 98%). Further comparison of genome completeness among these and other species from the *Rickettsiales* order showed a similar percentage of completeness for *A. platys*, in particular when compared with *A. phagocytophilum*. In addition, these results, as well as further phylogenetic analyses and a comparison of gene order and content, support the close relatedness between *A. phagocytophilum* and *A. platys*.

Despite the global similarity to other *Anaplasma* genomes, we found several potentially specific features in the *A. platys* genome. A notable difference is the apparent absence of *rbfA*, a gene encoding a 30S ribosome-binding factor involved in ribosome biogenesis and initiation of translation, also considered to be essential for *E. coli* cells to adapt to low temperature [23,24]. Exposure of bacteria to a low temperature shift triggers a specific pattern of gene expression known as the cold-shock response, characterized by the repression of heat-shock proteins. Expression of the *rbfA* gene in *E. coli* appears to be involved in the suppression of the cold-shock response, thus promoting a better adaptation to cold temperatures [25]. Exposure of an *rbfA* mutant to low temperature results in constitutive induction of the cold-shock response, accompanied by slower growth. The absence of this gene in *A. platys* potentially renders it more sensitive to cold environments, possibly determining the way it interacts with its tick vector. As there is no substantial evidence of vector competency of *Rhipicephalus sanguineus* for *A. platys* [26], it is tempting to speculate that the long-term survival of this pathogen is not possible in this vector due to its potential susceptibility to cold environments. However, further studies are required to confirm the absence of this gene in *A. platys* and its putative implication in vector transmission.

We also observed several differences associated with duplicated genes and those belonging to expanded gene families in the *A. platys* genome. In the *Anaplasma* genus, expanded gene families appear to evolve independently in different species, with several gene members that have either diverged into lineage-specific paralog groups in some species, while remaining as single-copy or becoming pseudogenes in other species. A typical example of this is the largest expanded gene family in these species, the *msp2* superfamily, encoding immunodominant outer membrane proteins. Within this family, genes from the *msp2/p44* subset appear to be selectively expanded in *A. phagocytophilum* and *A. platys*. In contrast, genes from the *omp* subset appear to be expanded in *A. marginale* and *A. ovis*. These subsets not only differ in gene content but also in the number of copies of individual members or paralog groups. For instance, in the subset expanded in *A. platys* and *A. phagocytophilum*, the overall number of copies, including also gene fragments and pseudogenes, appears to be higher in the latter. Pseudogenes from this superfamily are thought to be “stored” in the genome to help generate antigenic diversity through recombination [9].

Additional examples of expanded gene families are those encoding components of a type IV secretion system. This system is a multimeric complex that secrete proteins and DNA out into the host cell [27]. Subunits of these complex are encoded by at least 11 genes (*virB1*–*11*), all of which are considered important determinants of virulence in *Anaplasmataceae*. Expansion of gene families associated with the type IV secretion system is well documented in *Anaplasma*, with several reported copy number variations across species [12,28,29]. In the *A. platys* genome, we found duplication or expansion in the genes encoding VirB2, VirB8, VirB9, and VirB10. Genes encoding VirB6 are also arranged in four tandem copies with highly divergent sequences, resulting in their clustering into separate ortholog groups (OG_0396, OG_0397, OG_0398, and OG_0399).

Two more examples of expanded gene families with potentially species-specific members in the *A. platys* genome are those respectively encoding ankyrin repeat-containing proteins and proteins with predicted intrinsically disordered regions from ortholog groups OG_0007 and OG_0755. Ankyrin repeat-containing proteins are well-known virulence factors of intracellular bacteria, often mediating protein–protein interactions with host cell targets [30,31]. Differences among ankyrin repeat-containing proteins across *Anaplasma* species have been previously reported [12]. Since these proteins are effectors of the type IV secretion system [32], their expansion may be associated with the expansion of genes encoding components of this system, as discussed earlier. On the other hand, genes from OG_0007 and OG_0755 appear to form a tandem array of at least eight copies, with just one putative ortholog in *A. marginale* and *A. ovis* and two in *A. phagocytophilum*. We were not able to identify any additional functional domains or motifs in these genes, except for predicted intrinsically disordered regions. These are regions that do not adopt a defined secondary structure, which gives them higher structural flexibility, when compared to structured proteins [33]. Proteins with such regions are known to participate in interactions with other proteins and nucleic acids, often involved in cell signaling and adaptation to changing environments.

Very little is known about the genetic factors that determine the preference of *Anaplasma* species for particular cell types in their vertebrate hosts. *A. platys* invades platelets; however, there are no studies conducted to unravel its pathogenic mechanisms, including pathogen entry and cyclic thrombocytopenia. Platelets are known to interact with pathogens through their surface receptors and pattern recognition receptors TLR2 and TLR4 [5,34]. We did not find any evidence of the presence of known platelet-binding motifs in the *A. platys* genome, suggestive of its attachment to platelets. However, since many genetic differences among *Anaplasma* species appear to concentrate on expanded gene families, it is not illogical to speculate that host cell specificity may be determined by the interaction between lineage-specific members of these families and membrane receptors of the target cells. Future studies are required to understand not only host cell preference, but also the mechanisms involved in the survival and virulence of *A. platys*. The information presented in this study will pave the way to advance our knowledge of the evolutionary mechanisms conducive for species-specific colonization and survival of *Anaplasma* in unique niches in their hosts and vectors.

## 4. Materials and Methods

### 4.1. Ethical Approval Statement

The study was performed on the Caribbean island of Saint Kitts, located in the Lesser Antilles. The canine blood sample was obtained and stored from a client-owned patient presented to Ross University Veterinary Clinic (RUVC). Further research and investigations using this sample were conducted adhering to the Ross University School of Veterinary Medicine IACUC approved protocol (Protocol #17.04.21). All methods were carried out in accordance with relevant guidelines and regulations for the care and use of animals.

### 4.2. Blood Sampling and DNA Purification

The blood sample was obtained from a 5-year-old spayed female Blue Heeler mix dog presented to RUVC, Basseterre, Saint Kitts, West Indies, on July 26th, 2016. *A. platys* infection was confirmed by PCR as described previously [35]. The samples were submitted for routine CBC and blood chemistry analysis by the attending veterinarian, and the leftover samples were aliquoted and stored at –80 °C. We separated platelet-enriched plasma, buffy coat, and red blood cell (RBC) fractions after centrifugation at 1,000× g for 10 min. The platelet-enriched plasma was further centrifuged at 14,000× g to recover the pellet containing the platelets. The remaining RBC fraction was lysed using RBC lysis solution and centrifuged at 14,000× g for 5 min to recover the pellet. Both pellets and the buffy coat were pooled together and used for DNA extraction. DNA was extracted using the MasterPure™ DNA Purification Kit (Epicentre, Madison, WI, USA) and was stored at −80 °C until sequencing.

### 4.3. Whole Genome Sequencing

A total amount of 1 μg of genomic DNA was used as input material for library preparation. The sequencing library was generated with the DNA Library Prep kit for Illumina (New England Biolabs, Ipswich, MA, USA) following the manufacturer’s instructions. Briefly, the DNA sample was fragmented by sonication to a size of 350 bp, and then DNA fragments were ligated with the full-length adaptor for Illumina sequencing. Quality control of sequenced reads was performed with FastQC (version 0.11.9) (https://www.bioinformatics.babraham.ac.uk/projects/fastqc/), and low-quality regions were trimmed with trimmomatic (version 0.39) [36].

### 4.4. Genome Assembly

Illumina reads were initially mapped to the canine reference genome (CanFam3.1) [37] (downloaded from ftp://ftp.ncbi.nlm.nih.gov/genomes/) by using BWA–MEM (version 0.7.12) [38]. As a first step to minimize contamination with host DNA, all reads mapping to the canine reference genome were discarded. Unmapped reads were extracted from BWA–MEM alignments in BAM (binary alignment/map) format by using SAMtools (version 1.8) [39]. These unmapped reads were converted to the FASTQ format and were assembled with metaSPAdes (version 3.12) [18]. As a second step to minimize contamination, a sequence similarity search against the Genbank non-redundant (*nr*) database was performed for all scaffolds larger than 1 kb by using BLAST (blastn, version 2.9.0) [40]. Only those scaffolds with BLAST hits to existing *Anaplasma* sequences with an expect (*E*) value below 1 × 10^–10^ were retained in the assembly prior to subsequent steps.

In order to detect potential assembly errors, original Illumina reads were aligned against the newly generated scaffolds by using BWA–MEM. Read alignments were visually inspected in Artemis (version 17) [41] to detect regions in which the distribution of mapping pairs and the estimated distance between their members would suggest an assembly artifact. Scaffolds with potential artifacts were manually split into contigs at these points. The split contigs and the rest of the scaffolds were further oriented and placed into a single pseudochromosome by using ABACAS (version 1.3.1) [19] and the genome of *A. phagocytophilum* as a reference. Gaps of erroneous size, introduced during the contiguation step, were manually corrected or replaced by generic 100-bp gaps in cases where size could not be reliably estimated. The assembled pseudochromosome was compared to the *A. marginale* and *A. phagocytophilum* genomes by first creating comparison files with BLAST (tblastx, version 2.9.0) and then using these files to visualize the matches between each pair of genomes in the Artemis Comparison Tool (ACT) (version 17) [41].

### 4.5. Reference Genomes

The following genomes were used as a reference for the corresponding species in this study: *A. marginale* strain St. Maries (Genbank accession CP000030.1) [8], *A. ovis* strain Haibei (CP015994.2) [12], *A. phagocytophilum* strain HZ (CP000235.1) [9], and *E. chaffeensis* strain Arkansas (CP000236.1) [9]. Unless specified, explicit mention of the genome of these species throughout the article refers to these particular genomes.

### 4.6. Genome Annotation

Genes in the newly assembled pseudochromosome were annotated through two independent strategies, namely, the Prokka pipeline (version 1.14.3) [42], which was used to identify genes de novo, while RATT (version 1.0) [43] was used to tentatively transfer the gene models annotated in the *A. marginale* and *A. phagocytophilum* genomes. These two lines of evidence for gene models were manually combined through a three-way comparison of the genomes in ACT [41]. Putative pseudogenes were identified based on frameshifts or stop codons introduced in open reading frames, only in cases in which these variations were consistently confirmed by the majority of Illumina reads mapping to the corresponding locations.

### 4.7. Genome Completeness Analysis

Genome completeness was assessed with the BUSCO pipeline (v. 3.1.10) [20]. For this analysis, 23 genomes were considered (Table S1), including those from 15 species belonging to the *Anaplasmataceae* family (genera *Anaplasma*, *Wolbachia*, *Ehrlichia,* and *Neorickettsia*) and three from the *Rickettsiaceae* family (genus *Rickettsia*). We chose the genome of *Escherichia coli* strain K12 as the reference lineage, since this was the closest organism to *Anaplasma* among the available pre-defined reference lineages in the BUSCO pipeline. A different *E. coli* genome, that of strain O157:H7, and the genome of *Neisseria meningitidis* strain MC58 were also included in the analysis for comparative purposes, since no significant genome reduction has been previously reported for these species.

### 4.8. Phylogenetic Trees

The evolutionary position of *A. platys* strain S3 was explored by using concatenated amino acid sequences of conserved housekeeping genes and the nucleotide sequences of the 16S rRNA. For the tree built from protein sequences, we chose 14 species from the *Rickettsiales* order with fully annotated genomes available. To root the tree, *E. coli* and *N. meningitidis* were also included as outgroups. Amino acid sequences from proteins coded by 27 conserved genes extracted from these genomes (Table S2) were concatenated per species and aligned with ClustalW (version 2.1) [44]. The maximum likelihood (ML) tree was built by using PhyML (version 3.1) [45] with the Jones–Taylor–Thornton (JTT) model and 100 bootstrap replicates. For the tree of 16S rRNA sequences, four representative sequences of each *Anaplasma* species and *Ehrlichia chaffeensis* (Table S3) were aligned with MUSCLE (version 3.8.31) [46], and the ML tree was built by using PhyML with the Tamura–Nei (TN93) model and 500 bootstrap replicates.

### 4.9. Functional Analysis of Predicted Gene Models

OrthoMCL (version 2.0.9) [22] was used to cluster the gene models annotated in the *A. platys* genome together with those from *A. marginale*, *A. ovis*, *A. phagocytophilum,* and *E. chaffeensis* into putative ortholog groups, numbered consecutively and named with the “OG_” prefix. InterProScan (version 5) [47] was used to identify conserved domains and motifs. Functional categorization was performed by combining evidence from Clusters of Orthologous Groups (COG) inferences assigned by Prokka and Gene Ontology (GO) terms assigned by Interproscan. Metabolic reconstruction was performed with the BlastKOALA [48] and KEGG Mapper [49] tools (version 2.2) for searching against the Kyoto Encyclopedia of Genes and Genomes (KEGG). Briefly, BlastKOALA was first used to assign KEGG accession numbers (K numbers) to *A. platys* genes, and then the assigned K numbers were mapped against entire pathways by using KEGG Mapper.

### 4.10. Estimation of Gene Copy Numbers

SAMtools (depth command) was used to record the Illumina read depth per base along the entire *A. platys* chromosome. Median read depth was then estimated by removing local peaks higher or lower than one standard deviation of the mean. To compute the median read depth for each gene, the same procedure was used but additionally submitting a file with the corresponding annotations in BED format to SAMtools. The copy number for each gene was estimated by the ratio between its median read depth and the median read depth calculated for the entire chromosome. For genes within the same ortholog group, the total copy number was considered to be the sum of their individual estimates.

## Figures and Tables

**Table 1:** General features of selected *Anaplasma* genomes.

Feature	*A. marginale* Strain St. Maries	*A. ovis* Strain Haibei	*A. phagocytophilum* Strain HZ	*A. platys* Strain S3
Accession	CP000030.1	CP015994.2	CP000235.1	CP046391.1
Size (bp)	1,197,687	1,214,674	1,471,282	1,196,811
GC content (%)	49.76	48.86	41.64	45.57
Protein-coding genes	949	933	1,235	926 ^1^
Pseudogenes	20	44	117 ^2^	13
Ribosomal RNAs	3	3	3	3
Transfer RNAs	37	37	37	37

^1^ Total gene number, computed by combining annotated copies with those estimated from locally increased read depth. ^2^ This number comprises all the fragmented loci from to the *msp2* superfamily, regardless of their annotations in the genome.

## Supplementary Materials

The following are available online at https://www.mdpi.com/2076-0817/9/4/277/s1, Table S1: Genomes used in the genome completeness analysis, Table S2: Genes used for the tree of concatenated protein sequences, Table S3: Sequences used for the tree of 16 rRNA genes, Table S4: Ortholog groups shared among all the selected *Anaplasma* and *Ehrlichia* species, Table S5: Ortholog groups with no representative genes in at least one of the species considered, Table S6: Genes involved in metabolic pathways predicted to be complete in *A. platys*, Table S7: Genes suspected to be duplicated or expanded in the *A. platys* genome, Figure S1: Morulae in platelets of the blood extracted from the canine patient, compatible with *A. platys* infection, Figure S2: Potentially species-specific genes and/or duplicated genes with predicted intrinsically disordered regions, Figure S3: Conservation of gene order among representative *Anaplasma* species and *E. chaffeensis*, Figure S4: Neighbor-joining tree of genes from the *msp2* superfamily.
