# Supplementary material for: First Whole Genome Sequence of Anaplasma platys, an Obligate Intracellular Rickettsial Pathogen of Dogs
Source: Pathogens. 2020 Apr 10;9(4):277. doi: 10.3390/pathogens9040277 (PMC7238063; doi:10.3390/pathogens9040277)
Supplement: Supplementary file 1 [file pathogens-09-00277-s001.zip › Figure S3.pdf]

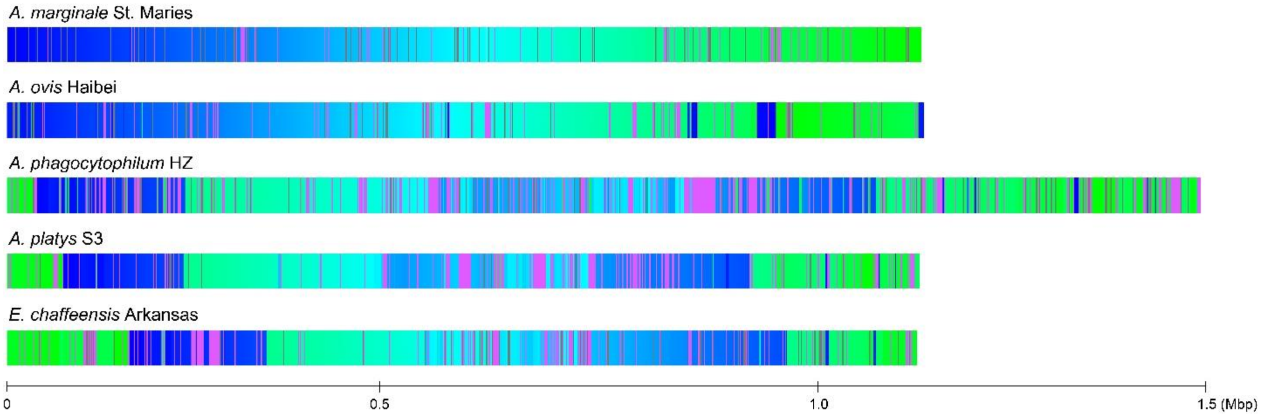

Figure S3. Conservation of gene order among representative *Anaplasma* species and *E. chaffeensis*. A color in a green-blue gradient was assigned to each ortholog group, ordered by the genes in the *A. marginale* genome. These colors were then plotted along the sequence of each genome according to their respective gene order. Potentially species-specific genes are highlighted in purple.
