# Supplementary material for: First Whole Genome Sequence of Anaplasma platys, an Obligate Intracellular Rickettsial Pathogen of Dogs
Source: Pathogens. 2020 Apr 10;9(4):277. doi: 10.3390/pathogens9040277 (PMC7238063; doi:10.3390/pathogens9040277)
Supplement: Supplementary file 1 [file pathogens-09-00277-s001.zip › Figure S4.pdf]

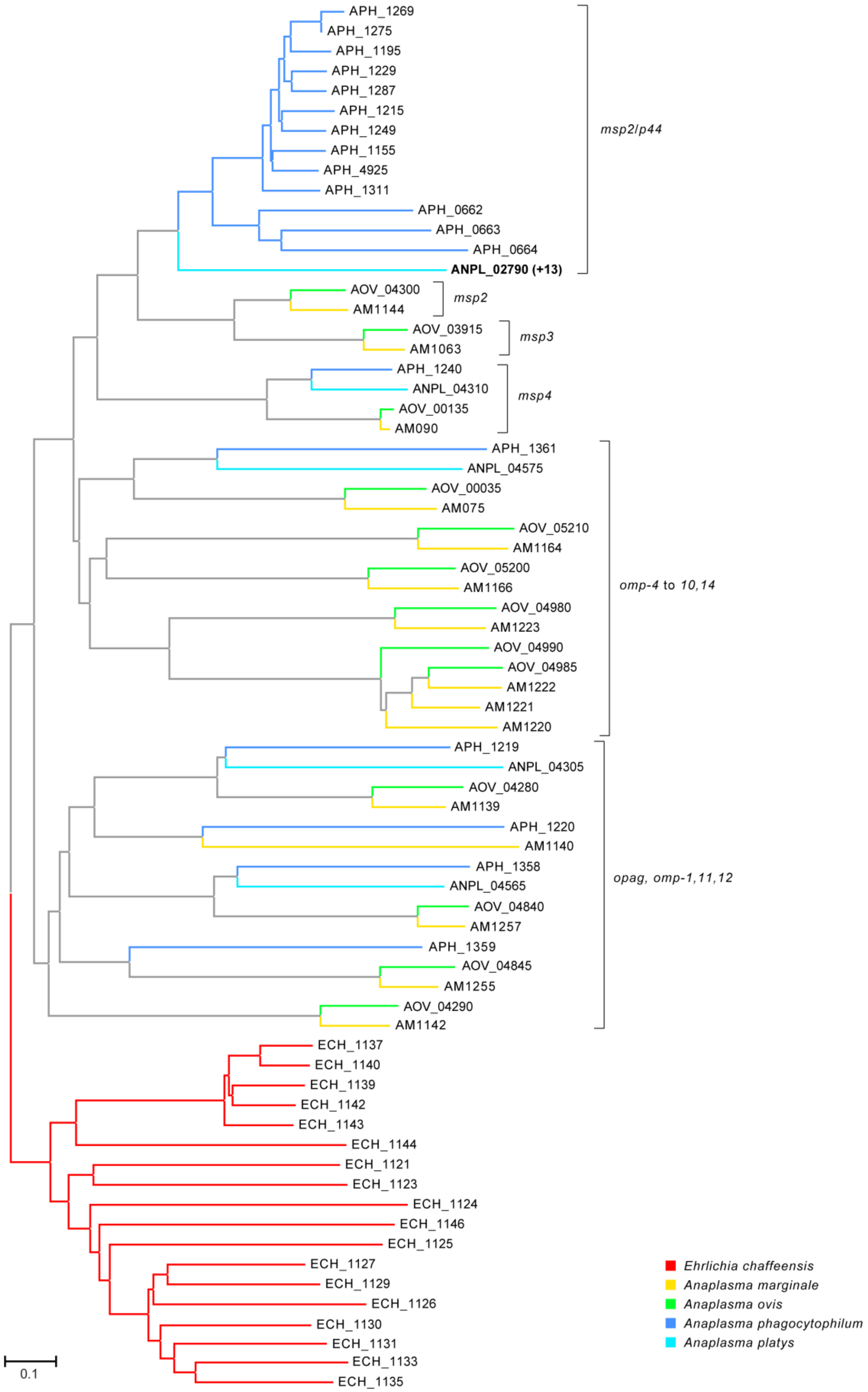

APH_1269
APH_1275
APH_1195
APH_1229
APH_1287
APH_1215
APH_1249
APH_1155
APH_4925
APH_1311
APH_0662
APH_0663
APH_0664
ANPL_02790 (+13)
msp2/p44
AOV_04300
AM1144
msp2
AOV_03915
AM1063
msp3
APH_1240
ANPL_04310
AOV_00135
AM090
msp4
APH_1361
ANPL_04575
AOV_00035
AM075
AOV_05210
AM1164
AOV_05200
AM1166
AOV_04980
AM1223
AOV_04990
AOV_04985
AM1222
AM1221
AM1220
omp-4 to 10,14
APH_1219
ANPL_04305
AOV_04280
AM1139
APH_1220
AM1140
APH_1358
ANPL_04565
AOV_04840
AM1257
APH_1359
AOV_04845
AM1255
AOV_04290
AM1142
opag, omp-1,11,12
ECH_1137
ECH_1140
ECH_1139
ECH_1142
ECH_1143
ECH_1144
ECH_1121
ECH_1123
ECH_1124
ECH_1146
ECH_1125
ECH_1127
ECH_1129
ECH_1126
ECH_1130
ECH_1131
ECH_1133
ECH_1135
0.1
Ehrlichia chaffeensis
Anaplasma marginale
Anaplasma ovis
Anaplasma phagocytophilum
Anaplasma platys

Figure S4. Neighbor-Joining tree of genes from the *msp2* superfamily. Genes potentially encoding members of the *msp2* superfamily were selected from the genomes of *A. marginale, A. ovis, A. phagocytophilum, A. platys* and *E. chaffeensis*. A preliminary alignment was performed with ClustalW to identify fragmented genes and putative pseudogenes, all of which were discarded. Remaining sequences were aligned with MUSCLE and the alignment was manually edited to remove hypervariable regions and those with more than 90% of gaps. The tree was built with the Neighbor-Joining method and rooted with the *E. chaffeensis* branch.
