# Supplementary material for: First Whole Genome Sequence of Anaplasma platys, an Obligate Intracellular Rickettsial Pathogen of Dogs
Source: Pathogens. 2020 Apr 10;9(4):277. doi: 10.3390/pathogens9040277 (PMC7238063; doi:10.3390/pathogens9040277)
Supplement: Supplementary file 1 [file pathogens-09-00277-s001.zip › Table S1.pdf]

Table S1. Genomes used in genome completeness comparisons.

Species	Strain/Isolate	Accession
Anaplasma centrale	Israel	NC_013532.1
A. marginale	Florida	NC_012026.1
A. marginale	St. Maries	CP000030.1
A. ovis	Haibei	CP015994.2
A. phagocytophilum	HZ	CP000235.1
A. phagocytophilum	JM	NC_021880.1
A. platys	S3	CP046391.1
Ehrlichia chaffeensis	Arkansas	CP000236.1
E. canis	Jake	NC_007354.1
E. canis	YZ-1	NZ_CP025749.1
E. ruminantium	Gardel	NC_006831.1
E. ruminantium	Welgevonden	NC_005295.2
Escherichia coli	O157: H7 Sakai	NC_002695.2
Neisseria meningitidis	MC58	NC_003112.2
Neorickettsia sennetsu	Miyayama	NC_007798.1
Rickettsia conorii	Malish 7	NC_003103.1
R. prowazekii	Madrid E	NC_000963.1
R. typhi	Wilmington	NC_006142.1
Wolbachia endosymbiont of *B. malayi*		NC_006833.1
W. endosymbiont of *D. melanogaster*		NC_002978.6
