# Supplementary material for: First Whole Genome Sequence of Anaplasma platys, an Obligate Intracellular Rickettsial Pathogen of Dogs
Source: Pathogens. 2020 Apr 10;9(4):277. doi: 10.3390/pathogens9040277 (PMC7238063; doi:10.3390/pathogens9040277)
Supplement: Supplementary file 1 [file pathogens-09-00277-s001.zip › Table S3.pdf]

Table S3. Sequences used for the tree of 16 rRNA genes.

Genbank Accession	Species	Strain/Isolate
U03775.1	*Anaplasma bovis*	-
KY425447.1	*Anaplasma bovis*	Y258
HM131217.1	*Anaplasma bovis*	Hiroshima-Z27
MF289479.1	*Anaplasma bovis*	FL17
AF414868.1	*Anaplasma centrale*	Vaccine
KU686784.1	*Anaplasma centrale*	Uganda KT5
JQ839010.1	*Anaplasma centrale*	C4B
ACIS_RS04170*	*Anaplasma centrale*	Israel
AF536828.1	*Anaplasma platys*	Okinawa
EU439943.1	*Anaplasma platys*	Gigio
MF289478.1	*Anaplasma platys*	YY36
ANPL_03590*	*Anaplasma platys*	S3
EF587237.1	*Anaplasma ovis*	CGX
JQ917906.1	*Anaplasma ovis*	XG53
JQ917893.1	*Anaplasma ovis*	WYG26
AOV_00895*	*Anaplasma ovis*	Haibei
AF309866.1	*Anaplasma marginale*	Virginia
JQ839012.1	*Anaplasma marginale*	C6A
AJ633048.1	*Anaplasma marginale*	Lushi
AM_RS01050*	*Anaplasma marginale*	St. Maries
AY527214.1	*Anaplasma phagocytophilum*	Strong
MN227385.1	*Anaplasma phagocytophilum*	AAIK4
NR_044762.1	*Anaplasma phagocytophilum*	Webster
APH_RS03965*	*Anaplasma phagocytophilum*	HZ
MH762074.1	*Anaplasma capra*	AK-Rm-309
LC432126.1	*Anaplasma capra*	KWD-35
MG869594.1	*Anaplasma capra*	XA143
KJ700627.1	*Anaplasma capra*	A60
AF147752.2	*Ehrlichia chaffeensis*	-
KX505292.1	*Ehrlichia chaffeensis*	X1
U60476.1	*Ehrlichia chaffeensis*	Sapulpa
ECH_RS03785*	*Ehrlichia chaffeensis*	Arkansas

* Gene ID from the corresponding annotated genome (see Table S1).
