# Supplementary material for: First Whole Genome Sequence of Anaplasma platys, an Obligate Intracellular Rickettsial Pathogen of Dogs
Source: Pathogens. 2020 Apr 10;9(4):277. doi: 10.3390/pathogens9040277 (PMC7238063; doi:10.3390/pathogens9040277)
Supplement: Supplementary file 1 [file pathogens-09-00277-s001.zip › Figure S2.pdf]

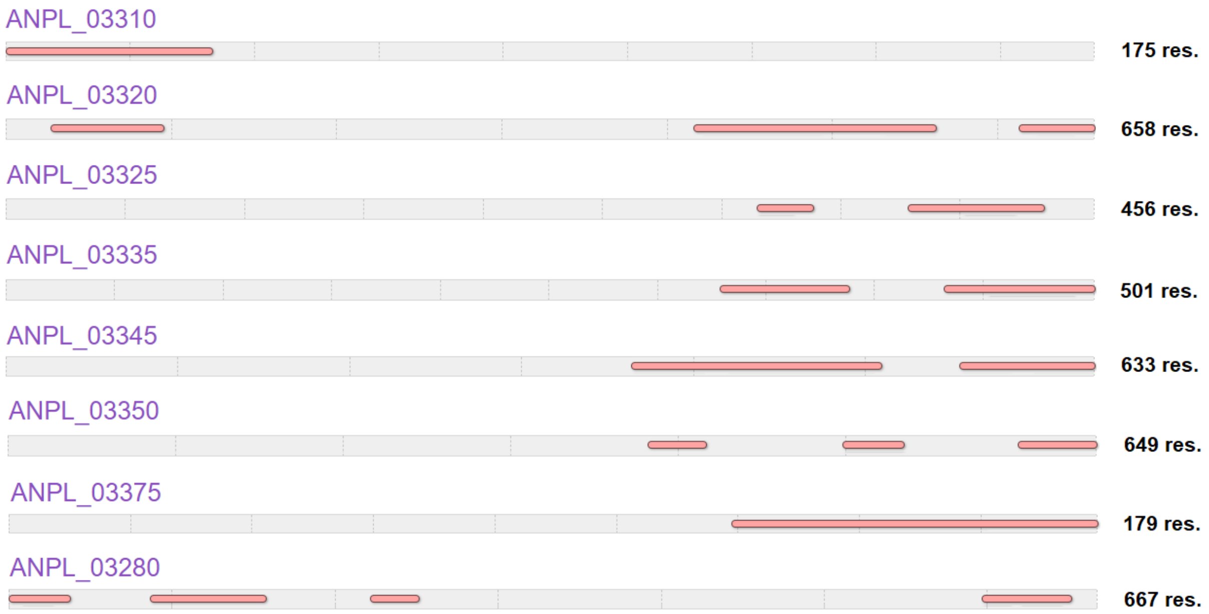

Figure S2. Potentially species-specific genes and/or duplicated genes with predicted intrinsically disordered regions. Regions predicted as intrinsically disordered by InterProScan are colored in pink along the sequence of the corresponding proteins.
